# Influence of Organ‐Specific Extranodal Involvement on Survival Outcomes in Stage IV Diffuse Large B‐Cell Lymphoma

**DOI:** 10.1002/cam4.70565

**Published:** 2024-12-31

**Authors:** Tong‐Yoon Kim, Tae‐Jung Kim, Eun Ji Han, Gi June Min, Sung‐Soo Park, Silvia Park, Jae‐Ho Yoon, Sung‐Eun Lee, Byung‐Sik Cho, Ki‐Seong Eom, Yoo‐Jin Kim, Hee‐Je Kim, Seok Lee, Chang‐Ki Min, Jong‐Wook Lee, Youngwoo Jeon, Seok‐Goo Cho

**Affiliations:** ^1^ Department of Hematology, Yeouido St. Mary's Hospital, College of Medicine The Catholic University of Korea Seoul Korea; ^2^ Lymphoma and Cell Therapy Research Center, Yeouido St. Mary's Hospital, College of Medicine The Catholic University of Korea Seoul Korea; ^3^ Department of Hospital Pathology, Yeouido St. Mary's Hospital, College of Medicine The Catholic University of Korea Seoul Korea; ^4^ Division of Nuclear Medicine, Department of Radiology, Yeouido St. Mary's Hospital, College of Medicine The Catholic University of Korea Seoul Korea; ^5^ Department of Hematology, Seoul St. Mary's Hospital, College of Medicine The Catholic University of Korea Seoul Korea

**Keywords:** autologous hematopoietic stem cell transplantation, diffuse large B‐cell lymphoma, extranodal involvement, positron emission tomography

## Abstract

**Background:**

The prognostic significance of extranodal sites in stage IV diffuse large B‐cell lymphoma (DLBCL) remains uncertain, making it challenging to select appropriate treatment strategies for individual patients. In this study, we aimed to evaluate the influence of different extranodal sites on prognosis in young patients with stage IV DLBCL who achieved complete remission (CR) following initial chemo‐immunotherapy and to explore the potential of autologous hematopoietic stem cell transplantation (ASCT) as a consolidation treatment for specific patient subgroups.

**Methods:**

We retrospectively reviewed data from 119 patients with DLBCL aged < 60 years who achieved CR after chemo‐immunotherapy between 2008 and 2020. Patient survival rates were analyzed in correlation with different extranodal sites using univariate and multivariate models. Additionally, we assessed the effect of ASCT on 5‐year progression‐free survival (PFS) and overall survival (OS) in patients with different extranodal sites involved.

**Study Design:**

A retrospective bicenter study.

**Results:**

Univariate analysis revealed a significant decrease in survival rates in patients with a Deauville score of 3 and those with extranodal DLBCL affecting the spleen, bone marrow, nasosinus, and liver. In multivariate analysis, only nasosinusal involvement remained a significant predictor of reduced OS. Patients with spleen involvement benefited significantly from ASCT in terms of 5‐year PFS and OS, whereas those with nasosinusal involvement did not demonstrate any survival advantage with ASCT.

**Conclusion:**

Our findings highlight the influence of specific extranodal sites on the prognosis of patients with stage IV DLBCL. The data indicate a clear need for precise patient stratification based on extranodal involvement for more effective treatment planning. Notably, patients with spleen involvement appear to benefit from ASCT, suggesting that this strategy could be useful in this subgroup. Further prospective studies are needed to confirm and incorporate these findings into clinical practice.

## 1 Introduction

Although the survival outcomes of patients with diffuse large B‐cell lymphoma (DLBCL) have improved after the introduction of rituximab, consolidation therapy for advanced‐stage DLBCL remains controversial owing to the high relapse rate. Whether end‐of‐treatment (EOT) positron emission tomography (PET)‐computed tomography (CT) with 18F‐fluorodeoxyglucose (FDG) can predict survival outcomes remains debatable [1, 2, 3]; however, observations have been made in patients who achieved complete remission (CR), even in stage IV DLBCL [4]. Despite consolidation radiation therapy (RT) being an effective option in advanced‐stage DLBCL [5, 6], selecting the optimal RT field is challenging because of the involvement of two or more sites or fragility owing to long‐term complications, notably in the gastrointestinal tract, pelvic bones, and kidneys [7, 8, 9]. Frontline autologous hematopoietic stem cell transplantation (ASCT) for high‐risk patients with DLBCL is suggested as an alternative [10].

ASCT is limited by treatment‐related mortality [11]. Therefore, ASCT has been used as a consolidation therapy for patients who achieved CR or partial remission (PR) in the chimeric antigen receptor (CAR) T‐cell therapy era [12, 13, 14]. However, considering the cumulative cycles of chemotherapy and the older age at diagnosis after relapse, frontline ASCT would be a good choice for consolidation therapy if the optimal patients could be identified.

Most DLBCLs originate in lymph nodes; however, approximately 40% are initially present in extranodal sites [15]. The most frequent site of origin is the gastrointestinal tract; however, many other organs, such as the testis, breast, and bone, are expected to have the worst outcomes even in stage I limited DLBCL [16]. Before the introduction of PET‐CT, identifying bone, spleen, and pleural involvement with contrast‐enhanced CT was challenging if measurable lesions were not evident. Currently, the corresponding organ uptake can be calculated using the Deauville score [17].

For the precise selection of high‐risk patients, we analyzed lymphomas involving extranodal sites on PET and attempted to identify optimal candidates for ASCT as consolidation therapy.

## 2 Methods

### 2.1 Patient Selection

This was a bicenter retrospective study. Patients (a) with DLBCL aged ≤ 60 years who were diagnosed in Seoul and Yeouido St. Mary's Hospitals between December 2008 and November 2020, (b) with de novo DLBCL without prior chemotherapy, (c) who underwent PET‐CT at diagnosis, interim, and EOT, (d) who were classified as Ann Arbor stage IV, (e) who were treated with a rituximab, cyclophosphamide, doxorubicin, vincristine, and prednisolone (R‐CHOP) regimen, and (f) who achieved CR at EOT PET‐CT were included. This study did not include patients with disease progression or those who died during chemotherapy.

### 2.2 Definitions of Diagnosis and Response Criteria

The diagnosis of DLBCL was established by three experienced senior pathologists at each center. The classification of germinal center B‐cell‐like (GCB) and non‐GCB subtypes was confirmed according to the Hans algorithm [18]. Staging and response were based on the Lugano classification [19]. To define CR, observation of complete regression of the measurable mass and a return to normal size on CT was necessary, along with a negative FDG‐PET scan, considering a Deauville score of 1–3 (details in Supporting Information). Bulky disease was defined as a lymphoma size larger than 7.5 cm, and a high maximum standardized uptake value (SUV_max_) was defined as an SUV of > 8.5. These cut‐off values were established based on previously reported associations with poor prognosis [20, 21].

### 2.3 Identification of Involved Extranodal Sites Using FDG‐PET and Clustering

The involved sites were confirmed based on FDG uptake on PET, and the accuracy of evaluation for size and location was verified using contrast‐enhanced CT. FDG uptake greater than the uptake intensity in the normal liver was considered the criterion for the involvement of extranodal sites in DLBCL (detailed in Supporting Information).

We used hierarchical clustering to identify extranodal areas that frequently co‐occurred. Extranodal involvement included in the clustering were as follows: spleen, bone marrow, nasosinus, head and neck glands, mediastinum or pericardium, lung or pleura, liver, gastrointestinal tract, retroperitoneum, breast, kidney, adrenal gland, genitals, axial bone, and skin or muscle. The final optimal cluster number for categorizing the patients with DLBCL was three, calculated by built‐in statistical program indices.

### 2.4 Treatment Protocol

The initial treatment for patients with DLBCL was 6 cycles of R‐CHOP chemotherapy [22]. Stem cell mobilization commenced after the end of the sixth R‐CHOP cycle. For mobilization, granulocyte colony‐stimulating factor (filgrastim, 10 g/kg) was administered 48 h after the sixth R‐CHOP infusion, and apheresis was performed when leukocyte and peripheral CD34 counts were elevated. In ASCT, we used reduced‐intensity BuMelTT protocols, which have been previously introduced (details in the Supporting Information) [10, 23].

In the non‐ASCT group, consolidation RT was administered to patients who received doses of 30–40 Gy, divided into 15–20 fractions. Those not fit for RT were considered the chemotherapy‐only group, with completion of the sixth cycle of R‐CHOP and regular follow‐ups.

### 2.5 Statistical Analysis

All categorical variables were analyzed using chi‐square analysis or Fisher's exact test, and all continuous variables were analyzed using Student's *t*‐test or the Mann–Whitney *U* test for intergroup comparisons. Overall survival (OS) was defined as the proportion of patients who survived until the end of the follow‐up period. Progression‐free survival (PFS) was assessed as the time from diagnosis to any event of death, treatment failure, and disease progression. OS and PFS were analyzed using the Kaplan–Meier survival curve, and log‐rank analysis was performed to compare data between different groups. Univariate and multivariate Cox proportional hazards models were constructed for OS using the clinical variables. The factors significantly affected by survival in the univariate analysis were used in a multivariable model for fitting. Statistical significance was set at a *p*‐value of < 0.05, and the *p*‐values reported are two‐sided.

Unsupervised analysis using hierarchical clustering was performed on our cohort by dividing the clusters. Extranodal involvement was evaluated in the spleen, bone marrow, nasosinus, head and neck glands, mediastinum or pericardium, lung or pleura, liver, gastrointestinal tract, retroperitoneum, breast, kidney, adrenal gland, genitals, axial bone, and skin or muscle. The number of clusters explored using the ward D2 method with the parameter package NbClust [24] ranged from 2 to 6 with binary distance, and the optimal number of clusters was chosen according to the following measures: the maximum value of the index (Calinski–Harabasz and Scott and Symons) and the maximum difference between the hierarchy levels of the index (Milligan and Cooper, Hartigan, and Friedman–Rubin). All statistical analyses were conducted using R software version 4.0.2 (R Foundation for Statistical Computing, 2017).

## 3 Results

### 3.1 Patient Characteristics

**FIGURE 1:**
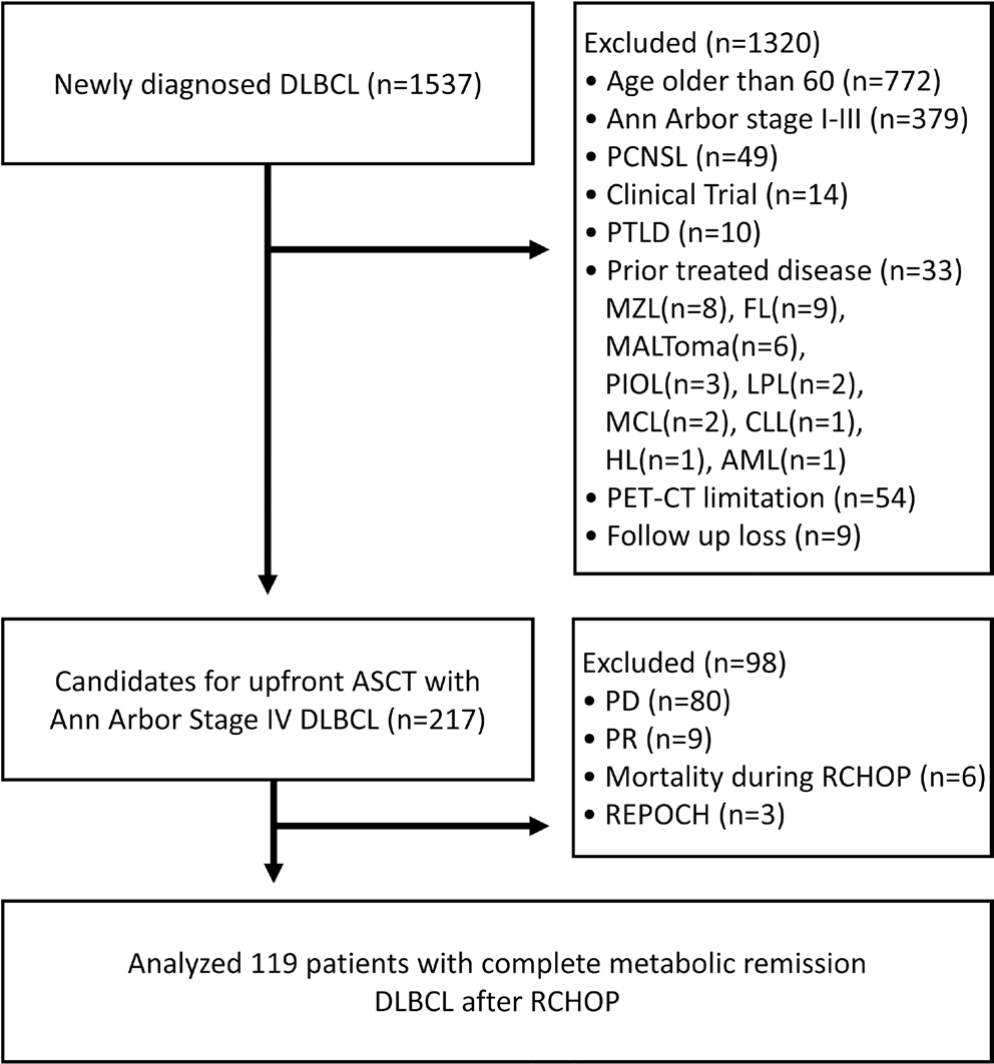
Flowchart of patient selection. AML, acute myeloid leukemia; CLL, chronic lymphocytic leukemia; DLBCL, diffuse large B‐cell lymphoma; FL, follicular lymphoma; HL, Hodgkin's lymphoma; LPL, lymphoplasmacytic lymphoma; MCL, mantle cell lymphoma; MZL, marginal zone lymphoma; PCNSL, primary central nervous system lymphoma; PD, progressive disease; PET‐CT, positron emission tomography‐computed tomography; PIOL, primary intraocular lymphoma; PR, partial remission; R‐CHOP, rituximab, cyclophosphamide, doxorubicin, vincristine, and prednisolone; REPOCH, rituximab, etoposide, prednisolone, doxorubicin, cyclophosphamide, and vincristine.

**FIGURE 2:**
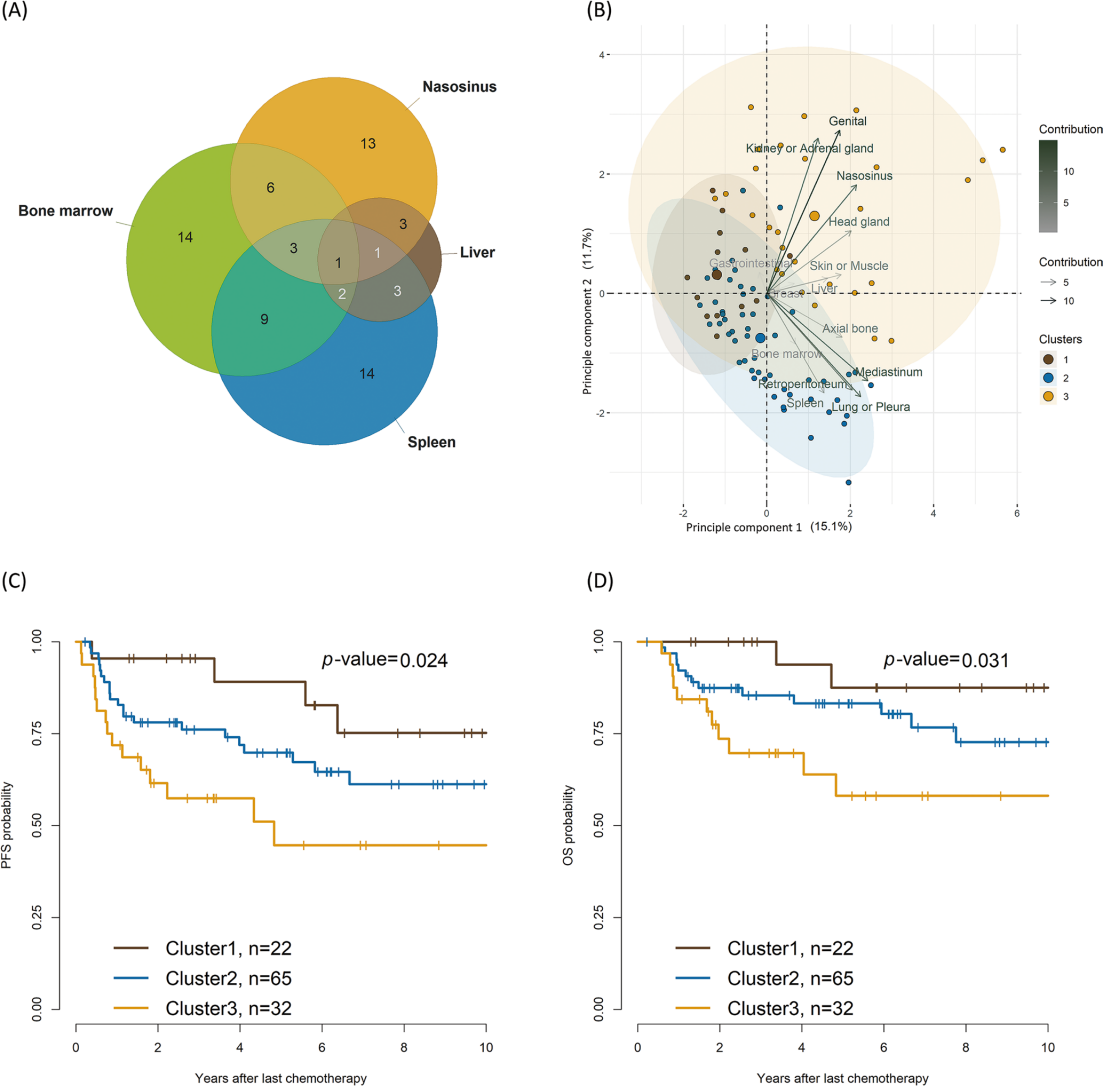
Distribution of extranodal sites in patients with stage IV diffuse large B‐cell lymphoma. (A) Venn diagram of involved organs with unfavorable overall survival in univariate analysis. (B) Biplot based on principal component analysis. The relative contributions of the involved sites on the *x* and *y* axes are indicated by an arrow with a gradient. Patients in three clusters categorized using a hierarchical clustering algorithm are shown as points. (C) Kaplan–Meier survival curve showing progression‐free survival of the three clusters, and (D) Kaplan–Meier survival curve showing overall survival of the three clusters.

Of the 1537 patients with DLBCL, 119 patients at stage IV with extranodal involvement who had achieved CR by the sixth cycle of R‐CHOP were eligible for the study; the detailed study design is shown in Figure 1. With a median follow‐up of 4.7 (range, 0.2–12.5) years, 37 patients underwent upfront ASCT; 82 patients in the non‐ASCT group underwent consolidation RT (*n* = 17) or observation after chemotherapy (*n* = 65). In the total cohort, 5‐year PFS and OS were 67.4% and 77.6%, respectively (Figure 2).

The baseline characteristics of the patients are summarized in Table 1. The median age at diagnosis was 51.5 (range, 45–57) and 48.0 (range, 39–55) years in the non‐ASCT and ASCT groups, respectively. No significant differences were found between the groups concerning age at diagnosis, sex, GCB type, or International Prognostic Index scores. Furthermore, the involved sites were not different between the groups.

**TABLE 1:** Patient characteristics.

Variable	Non‐ASCT (*N* = 82)	ASCT (*N* = 37)	*p*
Age at diagnosis, years (range)	51.5 (19–60)	48 (19–60)	0.097
Sex, female, *N* (%)	38 (46.3)	12 (32.4)	0.222
GCB type, *N* (%)	28 (34.1)	11 (29.7)	0.792
IPI score high, *N* (%)	21 (25.6)	14 (37.8)	0.255
Interim PET Deauville score 3 or 4	24 (29.3)	13 (35.1)	0.67
End‐of‐treatment PET Deauville score			0.543
1	53 (64.6)	20 (54.1)	
2	21 (25.6)	12 (32.4)	
3	8 (9.8)	5 (13.5)	
SUV_max_ (> 18), *N* (%)	23 (28)	15 (40.5)	0.254
Bulky disease (≥ 7.5 cm), *N* (%)	17 (20.7)	12 (32.4)	0.252
Spleen involvement, *N* (%)	26 (31.7)	11 (29.7)	0.999
Bone marrow involvement, *N* (%)	25 (30.5)	10 (27.0)	0.868
Nasosinusal involvement, *N* (%)	19 (23.2)	12 (32.4)	0.401
Mediastinum involvement, *N* (%)	24 (29.3)	12 (32.4)	0.895
Lung or pleura involvement, *N* (%)	15 (18.3)	6 (16.2)	0.988
Liver involvement, *N* (%)	8 (9.8)	2 (5.4)	0.664
Gastrointestinal involvement, *N* (%)	30 (36.6)	15 (40.5)	0.836
Breast involvement, *N* (%)	5 (6.1)	3 (8.1)	0.992
Kidney or adrenal gland involvement, *N* (%)	6 (7.3)	8 (21.6)	0.053
Genital involvement, *N* (%)	9 (11.0)	5 (13.5)	0.928
Axial bone involvement, *N* (%)	26 (31.7)	13 (35.1)	0.875
Skin or muscle involvement, *N* (%)	16 (19.5)	12 (32.4)	0.192

Abbreviations: ASCT, autologous hematopoietic stem cell transplantation; GCB, germinal center B‐cell‐like; IPI, International Prognostic Index; *N*, number; PET, positron emission tomography; SUV, standardized uptake value.

### 3.2 Impact of Involved Sites and Their Distribution on Patient Survival

In the total cohort, univariate analysis showed that patients with a Deauville score of 3 on EOT PET‐CT and DLBCL invasion in the spleen, bone marrow, nasosinus, and liver had inferior survival outcomes than those with noninvolvement. In the multivariate analysis, nasosinusal involvement showed inferior OS (hazard ratio [HR] = 2.69, 95% confidence interval = 1.22–5.91, *p* = 0.014) (Table S1).

Because advanced‐stage DLBCL at different extranodal sites co‐occurred frequently, we analyzed the impact of this co‐occurrence pattern on survival outcomes and the unfavorable sites involved using a Venn diagram (Figure 2A). We used a hierarchical clustering algorithm to categorize the involved sites into similar groups, utilizing a measurement‐based approach. The algorithm effectively partitioned the sites into three distinct groups. The three clusters were determined based on a voting process; a group of 2–6 clusters was explored using several measures included in the NbClust package. The best number of clusters was 4, and detailed indices for optimization are summarized in Table S2.

The biplot showed that cluster 1 was dominant with gastrointestinal involvement; cluster 2 was dominant with axial bone or mediastinum or pericardium involvement; and cluster 3 was dominant with nasosinusal, head and neck gland, kidney or adrenal gland, and genital organ involvement (Figure 2B). Detailed proportions of the extranodal involvement in each cluster are provided in Table S3. Some instances of site overlap were observed across the clusters, possibly because of the limitations of the applied clustering method or as a reflection of extranodal DLBCL characteristics, owing to which metastatic overlapping across clusters is unavoidable.

In the principal component analysis, principal component 1 (y‐axis) comprised mediastinum, lungs or pleura, and nasosinusal involvement. Principal component 2 (x‐axis) mostly comprised genital involvement, followed by kidney or adrenal, nasosinusal, lung, pleural, and spleen. The 5‐year PFS was 89.1%, 69.8%, and 44.7% in cluster 1, cluster 2, and cluster 3, respectively (cluster 1 vs. cluster 2, *p* = 0.2; cluster 2 vs. cluster 3, *p* = 0.07; cluster 3 vs. cluster 1, *p* = 0.01). The 5‐year OS was 87.5%, 83.2%, and 58.1% in cluster 1, cluster 2, and cluster 3, respectively (cluster 1 vs. cluster 2, *p* = 0.2; cluster 2 vs. cluster 3, *p* = 0.07; cluster 3 vs. cluster 1, *p* = 0.02; Figure 2C,D).

### 3.3 Impact of Involved Sites on ASCT Outcomes as Consolidation Therapy

**FIGURE 3:**
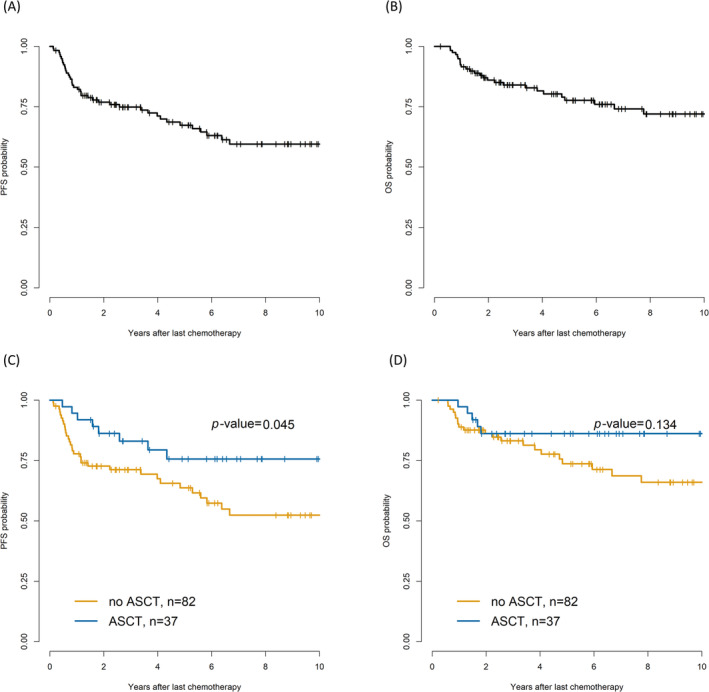
Progression‐free survival (PFS) (A) and overall survival (OS) (B) in patients with stage IV DLBCL. Impact of ASCT on PFS (C) and OS (D). ASCT, autologous hematopoietic stem cell transplantation; DLBCL, diffuse large B‐cell lymphoma.

The 5‐year PFS was 75.7% and 63.7% (*p* = 0.04) in the upfront ASCT and non‐ASCT groups (RT/observation), respectively (Figure 3A); however, the 5‐year OS rates were not significantly different (Figure 3B). Univariate analysis of OS showed that the HR for spleen involvement decreased from 3.43 to 0.64 in the ASCT group (*n* = 37) compared with that in the non‐ASCT group (*n* = 82). HR did not benefit from ASCT in cases of nasosinusal or liver involvement (Table 2). Among five patients who died from ASCT, one died because of hemorrhagic cystitis combined with cytomegalovirus infection; another died because of lymphoma progression.

**TABLE 2:** Univariate and multivariate analysis of overall survival by involved sites between the non‐ASCT and ASCT groups.

Univariate analysis				
Variable	Non‐ASCT		ASCT	
	HR, 95% CI	*p*	HR, 95% CI	*p*
GCB vs. non‐GCB	0.45 (0.15, 1.33)	0.147	0.52 (0.06, 4.69)	0.562
IPI score high–intermediate or high versus the other	1.05 (0.41, 2.71)	0.924	0.38 (0.04, 3.37)	0.383
Interim PET Deauville score 3 or 4 versus 1 or 2	1.48 (0.57, 3.86)	0.424	0.47 (0.05, 4.24)	0.504
EOT PET Deauville score 3 versus 1 or 2	4.61 (1.52, 14.0)	0.007	2.02 (0.23, 18.2)	0.529
SUV_max_ > 18 versus ≤ 18	1.29 (0.52, 3.21)	0.578	0.37 (0.04, 3.32)	0.375
Bulky disease ≥ 7.5 versus < 7.5 cm	0.31 (0.07, 1.35)	0.119	0.49 (0.05, 4.35)	0.518
Spleen involvement versus noninvolvement	3.43 (1.44, 8.19)	0.005	0.64 (0.07, 5.78)	0.695
Bone marrow involvement versus noninvolvement	2.45 (1.04, 5.78)	0.041	1.95 (0.32, 11.7)	0.467
Nasosinusal involvement versus noninvolvement	2.31 (0.95, 5.57)	0.063	9.55 (1.06, 85.6)	0.044
Mediastinum involvement versus noninvolvement	1.24 (0.50, 3.08)	0.641	3.57 (0.59, 21.4)	0.164
Lung or pleura involvement versus noninvolvement	1.75 (0.68, 4.52)	0.247	1.39 (0.16, 12.5)	0.767
Liver involvement versus noninvolvement	2.28 (0.76, 6.80)	0.14	13.5 (1.19, 154)	0.036
Gastrointestinal involvement versus noninvolvement	0.49 (0.18, 1.33)	0.16	1.04 (0.17, 6.21)	0.968
Breast involvement versus noninvolvement	NR (0.00, Inf)	0.998	NR (0.00, Inf)	0.999
Kidney or adrenal gland involvement versus noninvolvement	0.79 (0.11, 5.88)	0.816	1.01 (0.11, 9.05)	0.993
Genital involvement versus noninvolvement	1.04 (0.24, 4.46)	0.961	2.02 (0.23, 18.2)	0.529
Axial bone involvement versus noninvolvement	1.61 (0.66, 3.91)	0.291	0.47 (0.05, 4.24)	0.504
Skin or muscle involvement versus noninvolvement	1.68 (0.61, 4.63)	0.316	1.4 (0.23, 8.41)	0.711

Multivariate analysis				
	Non‐ASCT		ASCT	
EOT PET Deauville score 3 versus 1 or 2	5.47 (1.68, 17.8)	0.005		
Spleen involvement versus noninvolvement	4.19 (1.65, 10.6)	0.003		
Bone marrow involvement versus noninvolvement	2.16 (0.90, 5.20)	0.084		
Nasosinusal involvement versus noninvolvement	3 (1.18, 7.59)	0.02	7.76 (0.81, 74.7)	0.076
Liver involvement versus noninvolvement			5.15 (0.43, 62.1)	

Abbreviations: ASCT, autologous hematopoietic stem cell transplantation; CI, confidence interval; EOT, end‐of‐treatment; GCB, germinal center B‐cell‐like; HR, hazard ratio; IPI, International Prognostic Index; NR, not reached; *P*, *p*‐value; PET, positron emission tomography‐computed tomography; SUV, standardized uptake value.

**FIGURE 4:**
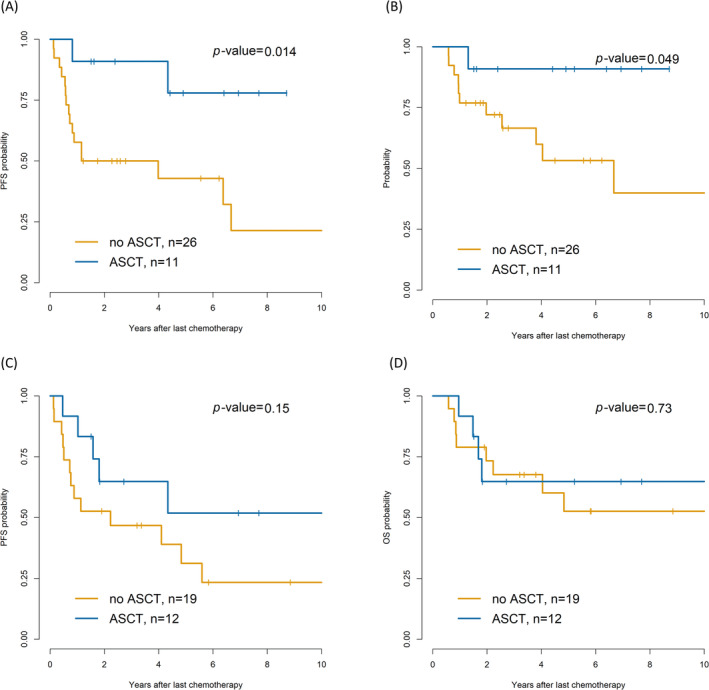
Subgroup analysis of progression‐free survival (PFS) (A) and overall survival (OS) (B) in patients with splenic involvement and of PFS (C) and OS (D) in patients with nasosinusal involvement. ASCT, autologous hematopoietic stem cell transplantation; OS, overall survival; PFS, progression‐free survival.

Among the 37 patients with splenic involvement, the ASCT group showed superior 5‐year PFS (77.9% vs. 21.4%, *p* = 0.014; Figure 4A) and OS (90.9% vs. 39.9%, *p* = 0.049; Figure 4B) compared with the non‐ASCT group. In contrast, the 31 patients with nasosinusal invasion showed no differences in PFS (*p* = 0.15) and OS (*p* = 0.73; Figure 4C,D). Among the patients with nasosinusal involvement, no notable differences in 5‐year PFS were observed between ASCT, RT, and observation (75.6% vs. 70.6% vs. 62.2%, *p* = 0.09).

## 4 Discussion

In this study, we evaluated the effect of extranodal involvement in chemosensitive stage IV DLBCL after introducing rituximab. Approximately 17%–23% of patients with advanced‐stage DLBCL with a complete metabolic response either experience relapse or die [1, 25]. Moreover, patients with DLBCL might experience relapse 5 years postdiagnosis (median 7.4 years), and the median age at relapse is 66 years [26]. Therefore, predicting clinical outcomes becomes important. We suggest that the extent of extranodal site uptake [27] on PET and the type of organ involved are important.

The sites involved, such as the bone marrow [28, 29], liver [30], spleen [31], and nasosinuses [32], could have different effects on prognosis. Our study showed that nasosinusal involvement not only worsened the outcomes but also was not associated with improvements in consolidation therapy outcomes, either RT or ASCT. Among the 31 patients, six underwent RT, but did not experience any advantage compared with those who received chemotherapy, consistent with the findings of a previous report [32, 33]. The poor prognosis in nasosinusal lymphoma might be associated with co‐occurrence of MYD88^L265P^ and CD79B genetic features, which enable the tumors to evade immune surveillance [34, 35].

The relapse pattern in patients with nasal DLBCL was extranodal dissemination. The tumor skips the lymphatic organs and spreads outside the head and neck, a pattern similar to that in nasal natural killer/T‐cell lymphoma [36]. On the contrary, Takahashi et al. [37] showed that involvement of the Waldeyer ring was associated with better outcomes of rituximab combined treatment. These differences might arise from differences in study samples. Their study included patients with limited‐stage DLBCL and refractory status, while the current study focused on consolidation treatment for chemotherapy responders in advanced‐stage DLBCL.

Although the impact of extranodal involvement is relatively well‐described for Ann Arbor Stage I DLBCL [16], that for stage IV is complicated due to the co‐occurrence of lymphoma at various sites. To solve this complex problem, we determined possible clustering utilizing an unsupervised algorithm. Furthermore, clustering groups describe different gathering trends by each involved organ and their effects on survival outcomes: Cluster 1, which is mainly associated with gastrointestinal involvement, was associated with favorable outcomes after CR; Cluster 2, predominantly involving the mediastinum and axial bones, was associated with further relapse compared with that of Cluster 1; Cluster 3, extending far from center of the body (e.g., predominantly involving the nasosinusal region, head and neck glands, kidneys or adrenal glands, and genital organs) was associated with more relapses even after PET CR than other clusters. These high‐risk sites involved in DLBCL are possible candidates for additional consolidative therapy. No differences were observed in GCB type between the clusters, and there was a lack of additional molecular data for comparison (Table S2). Cluster 3 is usually associated with a high risk of CNS relapse [38]. CNS relapse did not significantly differ among the three cluster groups; however, PFS and OS did. This difference might be attributed to the fact that our cohorts included only patients who achieved CR after chemotherapy and the exclusion of refractory DLBCL cases. Furthermore, the difference implies that regardless of CNS relapse, other mechanisms might promote relapse.

Because of unfavorable ASCT outcomes due to treatment‐related toxicity [11, 39], its role has been limited to salvage treatment of relapsed DLBCL [13, 14, 40]. Salvage ASCT candidates are patients who do not experience relapse/progression within 1 year of the initial diagnosis [4]. This means that the optimal timing of ASCT until relapse occurs could be missed because the risk of treatment‐related death increases with age.

To overcome this obstacle, we selected optimal candidates for frontline ASCT based on the specific organs involved in DLBCL. Recent studies have mainly focused on the number or size of extranodal sites for poor prognosis. However, we hypothesized that the specific organ involvement could help identify inferior outcomes and that the impacts are different based on the organ involved. For example, antitumor agents cannot reach organs with poor blood supply and, consequently, do not affect lymphoma.

Most studies agree on the advantages of frontline ASCT in controlling disease; however, ASCT does not correlate with survival gains [39, 41]. In a study by Wen et al. in which OS improved [42], patients with CR and PR aged 60–65 years were included. We excluded PET‐positive patients because they are expected to have diverse outcomes, especially early relapse, and require additional chemotherapy. Only patients aged ≤ 60 years were included to ensure that the toxicity was tolerable. However, we did not observe a superior OS with ASCT compared to that with RT/observation. We hypothesized that other factors would have influenced previously reported outcomes. Our data could explain the different outcomes in previous studies because some sites were chemoresistant even with high chemotherapy doses with ASCT. In this study, ASCT provided an advantage for patients with splenic involvement, the reason for which remains unclear. A possible explanation could be that spleen‐derived regulatory T cells are disturbed by tumor cells, as reflected by PET‐CT uptake in the spleen, and thus, functional renewal and T‐cell antigen receptor diversification of regulatory T cells induced by ASCT could enhance the antitumor effect [43].

Another hypothesis is that distinct genomic alterations in lymphomas can predispose patients to tumor invasion of various organs. Some studies have shown different genomic landscapes in extranodal DLBCL; for instance, CNS or testicular DLBCL has the *MYD88* mutation [44, 45], and breast DLBCL shows t(14;18)(q32;q21) translocation involving *MALT1* and *IGH* [46]. Wright et al. [47] described lymphomas with a co‐occurrence of MYD88^L265P^ and CD79B mutation group that frequently spread to extranodal sites including the CNS, testis, and breast. The study was the first to cluster molecular data and correlate the extranodal sites involved; conversely, our study conducted clustering based on the sites involved.

In our study, GCB versus non‐GCB types did not differ among the three cluster groups. Further studies are needed to determine the relationship between lymphoma sites and the genomic landscapes in DLBCL [48]. Furthermore, bulky disease and higher SUV_max_ did not affect both PFS and OS, possibly due to refractory disease exclusion in the study design. Regarding bulky mass with premised CR status, our study observed no inferior outcomes. Recent treatment options, such as tafasitamab plus lenalidomide [49], bispecific antibodies [50], and anti‐CD19 CAR‐T cell therapies [51], could be helpful as first‐line chemotherapy in patients with high risk for relapse. Furthermore, these patients could be candidates for maintenance therapy after first‐line chemo‐immunotherapy [52].

Owing to the retrospective nature of our study, some limitations regarding the intention‐to‐treat analysis must be acknowledged. Further prospective studies specifically focusing on the involved sites are necessary to provide more comprehensive insights. We included only patients under 60 years of age to reduce treatment‐related mortality. Therefore, our results cannot be applied to older patients. Further studies are needed to demonstrate the pathological and genetic differences between extranodal sites. An additional limitation is the relatively modest sample size of each consolidation treatment group, with small numbers of patients with organ‐specific involvement, making it challenging to draw definitive conclusions. Therefore, we performed clustering to identify similar patterns with small numbers of patients in the subgroups.

In conclusion, we evaluated the clinical outcomes of extranodal stage IV DLBCL in patients ≤ 60 years of age who achieved CR with chemo‐immunotherapy. Different DLBCL sites had different survival outcomes even though the patients achieved CR on EOT PET‐CT. Furthermore, we propose frontline ASCT as an optimal modality for consolidation therapy, depending on the tumor site. Our data could be useful in designing clinical trials for novel agents for patients expected to be resistant to RT or ASCT.

## Author Contributions

**Tong‐Yoon Kim:** conceptualization (lead), formal analysis (lead), writing – original draft (lead). **Tae‐Jung Kim:** data curation (equal), writing – review and editing (equal). **Eun Ji Han:** data curation (equal), writing – review and editing (equal). **Gi June Min:** data curation (equal), writing – review and editing (equal). **Sung‐Soo Park:** data curation (equal), writing – review and editing (equal). **Silvia Park:** resources (equal), writing – review and editing (equal). **Jae‐Ho Yoon:** resources (equal), writing – review and editing (equal). **Sung‐Eun Lee:** resources (equal), writing – review and editing (equal). **Byung‐Sik Cho:** resources (equal), writing – review and editing (equal). **Ki‐Seong Eom:** formal analysis (equal), writing – review and editing (equal). **Yoo‐Jin Kim:** formal analysis (equal), writing – review and editing (equal). **Hee‐Je Kim:** formal analysis (equal), writing – review and editing (equal). **Seok Lee:** formal analysis (equal), writing – review and editing (equal). **Chang‐Ki Min:** formal analysis (equal), writing – review and editing (equal). **Jong‐Wook Lee:** formal analysis (equal), writing – review and editing (equal). **Youngwoo Jeon:** conceptualization (equal), formal analysis (equal), resources (equal), supervision (equal), writing – original draft (equal). **Seok‐Goo Cho:** conceptualization (equal), formal analysis (equal), resources (equal), supervision (equal), writing – original draft (equal).

## Ethics Statement

This study was approved by the Institutional Review Board and Ethics Committee of the Catholic Medical Center in South Korea (XC23RADI0045).

## Consent

The authors have nothing to report.

## Conflicts of Interest

The authors declare no conflicts of interest.

## Supporting information


Data S1.

## Data Availability

The data presented in this study are available on request from the corresponding author. The data are not publicly available owing to privacy and ethical reasons.
