# Supplementary material for: Influence of Organ‐Specific Extranodal Involvement on Survival Outcomes in Stage IV Diffuse Large B‐Cell Lymphoma
Source: Cancer Med. 2024 Dec 31;14(1):e70565. doi: 10.1002/cam4.70565 (PMC11686428; doi:10.1002/cam4.70565)
Supplement: Supplementary file 1 — Data S1. [file CAM4-14-e70565-s001.docx]

**Supplementary Appendix**

**Table of contents**

[Methods 2](#_Toc166317611)

[Definitions of diagnosis and response criteria 2](#_Toc166317612)

[Identification of extranodal involvement using FDG-PET and clustering 3](#_Toc166317613)

[Treatment protocol 4](#_Toc166317614)

[Results 5](#_Toc166317615)

[**Supplementary Table S1.** Univariate and multivariate analysis of overall survival by site involvement in patients with stage IV DLBCL. 5](#_Toc166317616)

[**Supplementary Table S2.** The best number of clusters chosen by each index. 6](#_Toc166317618)

[**Supplementary Table S3.** Distribution of lymphoma in the three subgroups of sites involved determined using hierarchical clustering algorithm. 7](#_Toc166317619)

[References 8](#_Toc166317620)

# Methods

## **Definitions of diagnosis and response criteria**

The diagnosis of diffuse large B-cell lymphoma (DLBCL) was established by three experienced senior pathologists at each center. The classification of germinal center B-cell-like (GCB) and non-GCB subtypes was confirmed using immunohistochemistry (IHC) for CD10, B-Cell Lymphoma 6 Protein, and multiple myeloma oncogene 1 antibody according to the Hans algorithm.^1^ The staging and response were based on the Lugano classification.^2^

To define complete remission (CR), it was necessary to observe complete regression of the measurable mass and a return to normal size on computed tomography (CT) imaging along with a negative 18F-fluorodeoxyglucose-positron emission tomography (FDG-PET) scan, considering a Deauville score of 1–3. Partial remission (PR) was achieved if at least a 25% reduction in the size of all lesions was observed on CT with positive FDG-PET findings and no new lesions. Progressive disease (PD) was defined as the appearance of newly developed lesions or an increase in lesion size of more than 20% compared with the sizes of pretreatment lesions. Relapse was defined based on the appearance of new lesions in patients who had achieved CR or disease progression in those who had shown PR.

## **Identification of extranodal involvement using FDG-PET and clustering**

We performed FDG-PET imaging 60 min after the intravenous administration of FDG (370–555 MBq). All patients fasted at least 8 h before imaging, and none had blood glucose levels greater than 180 mg/dL before injection of FDG. The involved sites were confirmed by FDG uptake on PET, and the accuracy of size and location was verified using contrast-enhanced computed tomography. Bone marrow involvement was confirmed using biopsy with IHC or positive FDG-PET. FDG uptake greater than the intensity of uptake in the normal liver and lesions that resolved in parallel with the nodal disease during treatment were considered the criteria for FDG uptake in the extranodal sites involved in DLBCL.

We used hierarchical clustering to identify extranodal areas that frequently co-occurred. Extranodal organs involved included in clustering were spleen, bone marrow, nasosinus, head and neck glands, mediastinum or pericardium, lung or pleura, liver, gastrointestinal tract, retroperitoneum, breast, kidney, adrenal gland, genitals, axial bone, and skin or muscle. The final optimal cluster number for dividing the patients with DLBCL was three, calculated based on built-in statistical program indices.

## **Treatment protocol**

The initial treatment for patients with DLBCL was six cycles of R-CHOP chemotherapy.^3^ After three cycles of treatment with rituximab, cyclophosphamide, doxorubicin, vincristine, and prednisolone (R-CHOP), we analyzed the response using computed tomography (CT) and FDG PET. If patients achieved CR or PR, additional cycles of R-CHOP were performed for a total of six cycles. For R-CHOP chemotherapy, rituximab was infused at 375 mg/m^2^ on day 1, cyclophosphamide at 750 mg/m^2^ on day 1, doxorubicin at 50 mg/m^2^ on day 1, vincristine at1.4 mg/m^2^, but no more than 2.0 mg in total on day 1, and oral prednisolone 60 mg on days 1 to 5 and then continued every 3 weeks. Stem cell mobilization was performed after the end of the last 6^th^ R-CHOP cycle. For mobilization, granulocyte colony-stimulating factor (filgrastim, 10 g/kg) was administered 48 h after the sixth R-CHOP infusion, and apheresis was performed when leukocyte and peripheral CD34 counts were elevated.

In the autologous stem cell transplantation, we used reduced-intensity BuMelTT protocols, including intravenous busulfan (2.4 mg/kg/day for 3 days; D-8 to D-6), melphalan (40 mg/m^2^/day for 2 days; D-5 and D-4), and thiotepa (200 mg/m^2^/day for 2 days; D-3 and D-2).^4,5^ The collected stem cells were infused 48 hours after the last chemotherapy.

In the non-autologous hematopoietic stem cell transplantation group, consolidative RT was performed in patients treated with 30–40 Gy dose divided into 15–20 fractions. Those not fit for radiotherapy were considered the chemotherapy-only group, with completion of the sixth cycle of R-CHOP and regular follow-ups.

# Results

## **Supplementary Table S1**. Univariate and multivariate analysis of overall survival by site involvement in patients with stage IV DLBCL.

|  | | Progression-free survival | | | | Overall survival | | | | |
| --- | --- | --- | --- | --- | --- | --- | --- | --- | --- | --- |
|  | Univariate | | | Multivariate | | | Univariate | | Multivariate | |
| Variable | HR, 95%  CI | | P | HR, 95%  CI | P | | HR, 95%  CI | P | HR, 95%  CI | P |
| GCB type vs. non-GCB | 0.58  (0.27, 1.21) | | 0.147 |  |  | | 0.48  (0.18, 1.28) | 0.144 |  |  |
| IPI score High or High–Intermediate | 1.06  (0.55, 2.06) | | 0.858 |  |  | | 0.77  (0.32, 1.83) | 0.549 |  |  |
| Interim PET Deauville score 3 or 4 vs. 1 or 2 | 1.12  (0.57, 2.21) | | 0.74 |  |  | | 1.06  (0.44, 2.53) | 0.897 |  |  |
| EOT PET Deauville score 3 vs. 1 or 2 | 1.78  (0.69, 4.57) | | 0.234 |  |  | | 3.6  (1.34, 9.68) | 0.011 | 2.44  (0.81, 7.36) | 0.113 |
| SUVmax >18 vs. ≤18 | 0.68  (0.34, 1.37) | | 0.28 |  |  | | 0.88  (0.38, 2.03) | 0.767 |  |  |
| Bulky disease ≥7.5 vs. <7.5cm | 0.39  (0.15, 1.00) | | 0.051 |  |  | | 0.35  (0.11, 1.17) | 0.089 |  |  |
| Spleen INV vs non-INV | 2.35  (1.26, 4.40) | | 0.007 | 1.82  (0.90, 3.66) | 0.094 | | 2.42  (1.11, 5.27) | 0.026 | 2.09  (0.89, 4.93) | 0.092 |
| Bone marrow INV vs non-INV | 2.05  (1.08, 3.86) | | 0.027 | 1.76  (0.90, 3.46) | 0.099 | | 2.43  (1.12, 5.27) | 0.024 | 2.17  (0.97, 4.86) | 0.059 |
| Nasosinus INV vs non-INV | 2.98  (1.60, 5.57) | | <0.001 | 2.84  (1.48, 5.45) | 0.002 | | 2.8  (1.29, 6.05) | 0.009 | 2.69  (1.22, 5.91) | 0.014 |
| Mediastinum INV vs non-INV | 2.03  (1.09, 3.81) | | 0.027 | 1.5  (0.76, 2.95) | 0.24 | | 1.53  (0.69, 3.37) | 0.293 |  |  |
| Lung or pleura INV vs non-INV | 1.39  (0.66, 2.92) | | 0.386 |  |  | | 1.7[0.72, 4.06] | 0.228 |  |  |
| Liver INV vs non-INV | 4.01  (1.84, 8.75) | | <0.001 | 2.69  (1.12, 6.45) | 0.027 | | 3.19  (1.20, 8.48) | 0.02 | 1.58  (0.50, 5.00) | 0.433 |
| Gastrointestinal INV vs non-INV | 0.67  (0.34, 1.32) | | 0.245 |  |  | | 0.58  (0.24, 1.38) | 0.215 |  |  |
| Breast INV vs non-INV | 0.27  (0.04, 1.94) | | 0.191 |  |  | | NR  (0.00, Inf) | 0.997 |  |  |
| Kidney or adrenal gland INV vs non-INV | 0.43  (0.10, 1.78) | | 0.245 |  |  | | 0.74  (0.18, 3.14) | 0.684 |  |  |
| Genital INV vs non-INV | 0.66  (0.20, 2.16) | | 0.496 |  |  | | 1.18  (0.36, 3.95) | 0.783 |  |  |
| Axial bone INV vs non-INV | 1.16  (0.61, 2.22) | | 0.652 |  |  | | 1.18  (0.53, 2.65) | 0.69 |  |  |
| Skin or muscle INV vs non-INV | 1.21  (0.59, 2.48) | | 0.596 |  |  | | 1.3  (0.55, 3.10) | 0.55 |  |  |

## ASCT, autologous hematopoietic stem cell transplantation; GCB, Germinal center B-cell like; IPI, International prognostic index; PET, Positron emission tomography–computed tomography; EOT, end of treatment; SUV, standardized uptake value; INV, Involvement; HR, hazard ratio; CI, confidence interval; P, p-value; NR, Not reached

## **Supplementary Table S2.** Best number of clusters chosen by each index.

| Number  of clusters | Number of criteria | Detailed criteria in NbClust package | Detailed criteria reference |
| --- | --- | --- | --- |
| 2 | 6 | CCC, Dunn, Duda, seudoT2, Beale, McClain, | Maximum value of the index (Sarle 1983, Dunn 1974)  Smallest $n_{c}$index>criticalValue (Duda and Hart 1973), Smallest $n_{c}$index<criticalValue (Duda and Hart 1973), $n_{c}$such that critical value of the index >= alpha (Beale 1969), Minimum value of the index (McClain and Rao 1975) |
| 3 | 9 | CH, Hartigan, Scott, TrCovW, Ball, Friedman, TraceW,  Marriot, Rubin | Maximum value of the index (Calinski and Harabasz 1974, Hartigan 1975), Maximum difference between hierarchy levels of the index (Scott and Symons 1971, Milligan and Cooper 1985, Ball and Hall 1965, Friedman and Rubin 1967), Maximum value of absolute second differences between levels of the index (Milligan and Cooper 1985), Maximum value of second differences (Marriot 1971), Minimum value of second differences (Friedman and Rubin 1967) |
| 4 | 1 | KL | Maximum value of the index (Krzanowski and Lai 1988) |
| 5 | 2 | Silhouette,  Ratkowsky, | Maximum value of the index (Rousseeuw 1987, Ratkowsky and Lance 1978) |
| 6 | 5 | PtBiserial,  Cindex,DB,  Sdindex, SDbw | Maximum value of the index (Milligan 1980, 1981),  Minimum value of the index (Hubert and Levin 1976, Davies and Bouldin 1979, Halkidi et al. 2000, Halkidi and Vazirgiannis 2001) |

##

## **Supplementary Table S3.** Distribution of lymphoma in the three subgroups of sites involved determined using hierarchical clustering algorithm.

| Variable involved (N, %) | C1,  N=22 | C2,  N=65 | C3,  N=32 | C1 vs. C2  *p*-value | C2 vs. C3  *p*-value | C1 vs. C3  *p*-value |
| --- | --- | --- | --- | --- | --- | --- |
| GCB | 9 (40.9) | 22 (33.8) | 8 (25.0) | 0.734 | 0.514 | 0.348 |
| Spleen | 5 (22.7) | 23 (35.4) | 9 (28.1) | 0.404 | 0.627 | 0.898 |
| Bone marrow | 4 (18.2) | 23 (35.4) | 8 (25.0) | 0.215 | 0.424 | 0.796 |
| Nasosinus | 3 (13.6) | 3 (4.6) | 25 (78.1) | 0.339 | <0.001 | <0.001 |
| Head gland | 4 (18.2) | 9 (13.8) | 19 (59.4) | 0.883 | <0.001 | 0.006 |
| Mediastinum | 0 (0.0) | 26 (40.0) | 10 (31.2) | 0.001 | 0.538 | 0.011 |
| Lung or pleura | 1 (4.5) | 15 (23.1) | 5 (15.6) | 0.105 | 0.558 | 0.405 |
| Liver | 1 (4.5) | 3 (4.6) | 6 (18.8) | >0.999 | 0.06 | 0.265 |
| Gastrointestinal | 22 (100.0) | 16 (24.6) | 7 (21.9) | <0.001 | 0.965 | <0.001 |
| Retroperitoneum | 3 (13.6) | 22 (33.8) | 10 (31.2) | 0.124 | 0.979 | 0.245 |
| Breast | 1 (4.5) | 5 (7.7) | 2 (6.2) | 0.987 | >0.999 | >0.999 |
| Kidney and adrenal gland | 2 (9.1) | 2 (3.1) | 10 (31.2) | 0.565 | <0.001 | 0.112 |
| Genital | 1 (4.5) | 0 (0.0) | 13 (40.6) | 0.567 | <0.001 | 0.008 |
| Axial bone | 1 (4.5) | 28 (43.1) | 10 (31.2) | 0.002 | 0.368 | 0.04 |
| Skin or muscle | 0 (0.0) | 17 (26.2) | 11 (34.4) | 0.018 | 0.547 | 0.006 |

N, Number; C, Cluster; GCB, germinal center B-cell-like

# References

1. Hans CP, Weisenburger DD, Greiner TC, Gascoyne RD, Delabie J, Ott G, Müller-Hermelink HK, Campo E, Braziel RM, Jaffe ES, et al. Confirmation of the molecular classification of diffuse large B-cell lymphoma by immunohistochemistry using a tissue microarray. Blood. 2004;103(1):275-282. DOI: 10.1182/blood-2003-05-1545.

2. Cheson BD, Fisher RI, Barrington SF, Cavalli F, Schwartz LH, Zucca E, Lister TA; Alliance, Australasian Leukaemia and Lymphoma Group; Eastern Cooperative Oncology Group; European Mantle Cell Lymphoma Consortium; et al. Recommendations for initial evaluation, staging, and response assessment of Hodgkin and non-Hodgkin lymphoma: the Lugano classification. Journal of Clinical Oncology. 2014;32(27):3059-3067. DOI: [10.1200/JCO.2013.54.8800](https://doi.org/10.1200/jco.2013.54.8800).

3. Coiffier B, Lepage E, Brière J, Herbrecht R, Tilly H, Bouabdallah R, Morel P, Van Den Neste E, Salles G, Gaulard P, et al. CHOP chemotherapy plus rituximab compared with CHOP alone in elderly patients with diffuse large-B-cell lymphoma. New England Journal of Medicine. 2002;346(4):235-242. DOI: [10.1056/NEJMoa011795](https://doi.org/10.1056/nejmoa011795).

4. Yoon JH, Min GJ, Park SS, Jeon YW, Lee SE, Cho BS, Eom KS, Kim YJ, Lee S, Kim HJ, et al. Autologous hematopoietic cell transplantation using dose-reduced intravenous busulfan, melphalan, and thiotepa for high-risk or relapsed lymphomas. Bone Marrow Transplantation. 2019;54(2):330-333. DOI: [10.1038/s41409-018-0289-z](https://doi.org/10.1038/s41409-018-0289-z).

5. Yoon JH, Kim JW, Jeon YW, Lee SE, Eom KS, Kim YJ, Lee S, Kim HJ, Min CK, Lee JW, et al. Role of frontline autologous stem cell transplantation in young, high-risk diffuse large B-cell lymphoma patients. Korean Journal of Internal Medicine. 2015;30(3):362-371. DOI: [10.3904/kjim.2015.30.3.362](https://doi.org/10.3904/kjim.2015.30.3.362).
